# A maternally inherited 8.05 Mb Xq21 deletion associated with Choroideremia, deafness, and mental retardation syndrome in a male patient

**DOI:** 10.1186/s13039-017-0324-6

**Published:** 2017-06-14

**Authors:** Siying Liang, Nan Jiang, Shuo Li, Xiaohu Jiang, Dongyi Yu

**Affiliations:** 1 Genetic Testing Center, Qingdao Women and Children’s Hospital, Qingdao University, #217, Liaoyangxi Road, Qingdao, 266034 China; 2 Qingdao Women and Children’s Hospital, Qingdao University, Qingdao, China

**Keywords:** Xq21, Choroideremia, Deafness, Mental retardation, SNP arrays

## Abstract

**Background:**

Deletions in Xq21 cause various congenital defects in males including choroideremia, deafness and mental retardation, depending on their size and gene content. Until now only a limited number of patients with Xq21 deletions has been reported.

**Case presentation:**

Here we describe a 17-year-old male with choroideremia, deafness, and mental retardation syndrome. Using SNP arrays, an 8.05 Mb deletion in Xq21 was identified inherited from the apparently healthy mother. The deleted region harbors 12 OMIM genes, of which *POU3F4, CHM,* and *ZNF711* might have contributed to the patient’s phenotype including hearing loss, poor vision, and intellectual disability. Moreover, the patient’s mother exhibits a normal phenotype while carrying the same deletion, which is often observed in previous studies on female carriers in families with this syndrome.

**Conclusions:**

Our study confirms the causative effect between the Xq21 deletion in males and choroideremia, deafness and mental retardation.

## Background

X-chromosome deletions can result in serious congenital defects. Males seldom exhibit these defects, as deletions on the X chromosome are essentially lethal in male embryos discarded at an early embryonic stage. Even though high-resolution SNP array analysis has been performed to identify small deletions on the X chromosome, the clinical significance of Xq21 deletions in males is still poorly understood. It has been shown that a contiguous gene Xq21 deletion, including *POU3F4, CHM* and *ZNF711* genes, could result in choroideremia, deafness, and mental retardation syndrome. This deletion produces degeneration of the choriocapillaris, the retinal pigment epithelium, and the photoreceptor of the eye, as well as inner ear abnormality with progressive mixed hearing loss and intellectual disability. In the last decades, only six cases with Xq21 deletions have been reported [1–3]. Despite the severe symptoms exhibited by male probands, most female carriers are non-symptomatic or express only a mild phenotype [2].

Here we describe the case of a 17-year-old male with mental retardation, choroideremia, hearing impairment, cochlear deformity, and facial anomalies. Analysis by SNP array, identified an 8.05 Mb contiguous gene deletion in Xq21 harboring *POU3F4, CYLC1, RPS6KA6, HDX, APOOL, SATL1, ZNF711, POF1B, CHM, DACH2, KLHL4*, and *CPXCR1* genes. The same deletion was identified in the apparently healthy mother. Our data contribute to further understanding the correlation between Xq21 deletions and the abnormal phenotypes. Furthermore, we present all the Xq21 deletion cases previously described in order to review the matter of genotype-phenotype correlations.

## Case presentation

**Fig. 1:**
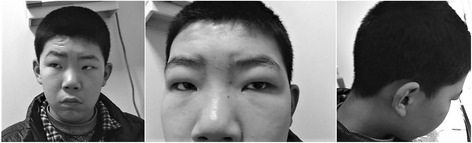
Facial dysmorphism of the patient, including narrow forehead, slightly drooping eyelids, short palpebral fissures and prominent ears

**Fig. 2:**
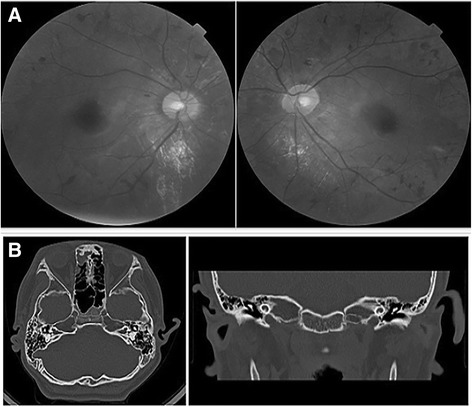
**a** Ophthalmoscopy photos of the patient showed the partial degeneration of the choroid in both eyes. **b** High-resolution CT imaging showed the cochlea deformity of the patient, including the short base turn of the cochlea, unclear division between the second turn and the apical turn of the cochlea and the absence of the modiolus

The patient was born by spontaneous delivery after an uneventful pregnancy (birth weight 3.350 kg and occipitofrontal circumference 35.5 cm) to phenotypically normal parents. He showed global development delay since infancy. He learned to speak at 18 months old and to walk at 2.5 years old. During childhood, he showed severe learning impairment and social disabilities. However, he was able to react to verbal words and to carry out brief communications. He has a narrow forehead, slightly drooping eyelids, short palpebral fissures, and prominent ears (Fig. 1). His IQ level is below 40, according to the Wechsler Intelligence Scale test, and his general cognitive ability is also low. His vision test result was out of the normal scale, indicating poor sight. His hearing test showed moderate hearing impairment with 60 dB of hearing loss. Ophthalmoscopy showed partial degeneration of the eye choroid (Fig. 2a). High resolution CT (HRCT) imaging showed that the base turn of the cochlea was shortened, the second turn and the apical turn were merged or unclearly divided, and the modiolus was absent, which evidently supported for the inner ear malformation (Fig. 2b). The patient’s mother also underwent the tests described above. However, her results were normal and there was no evidence suggesting that she might exhibit choroideremia, or hearing impairment in the future.

## Methods

Peripheral blood samples of the patient and his mother were used for genomic DNA extraction. Briefly, genomic DNA was isolated from whole blood using DNA Blood Mini Kit (Qiagen, Germany). Chromosomal microarray analysis was performed using Cytoscan 750 K chip, (Affymetrix, USA) according to manufacturer’s instructions. CMA was performed with CytoScan 750 K array (Affymetrix, Santa Clara, CA, USA) according to the manufacturer’s recommendations. The platform is composed of 550,000 non-polymorphic CNV probes and more than 200,000 SNP probes with an average resolution of 100 kb. The data was analyzed using chromosome analysis software ChAS (Affymetrix, USA). Primers flanking the deleted fragment, *POU3F4*and *CPXCR1*, were designed using Primer 5.0 (Premier, Canada) and were used to validate the deletion by PCR and agarose electrophoresis. Data collection were performed via searching in DECIPHER database (https://decipher.sanger.ac.uk/) and ISCA (https://www.clinicalgenome.org/) in order to identify cases of patients carrying similar deletions [4].

## Results

**Fig. 3:**
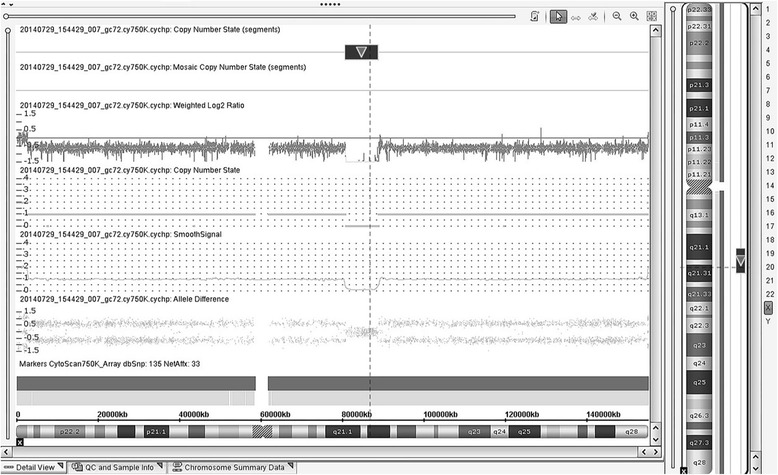
Detected by SNP arrays analysis, an 8.05 Mb deletion on the long arm of the X chromosome at Xq21 in the patient

SNP array analysis detected an 8.05 Mb deletion in the long arm of the X chromosome at Xq21.1-21.31 (Fig. 3) in both the patient and his mother. The genomic position of this deletion according to GRCh37/hg19 was Chr X: 80,817,978-88,868,979. The deleted region harbors 12 genes, including *POU3F4, CYLC1, RPS6KA6, HDX, APOOL, SATL1, ZNF711, POF1B, CHM, DACH2, KLHL4* and *CPXCR1*(Table 1). The PCR result confirmed the SNP array data.

**Table 1:** OMIM genes included in the deletion, protein products and function and disease caused

Gene	Protein	Protein function	Disease
POU3F4	POU class 3 homeobox 4	Inner ear development	Non-syndromic hearing loss
CYLC1	Cylicin, basic protein of sperm head cytoskeleton 1	Spermatid differentiation	None
RPS6KA6	Ribosomal protein S6 kinase A6	Activation of the mitogen-activated kinase cascade	None
HDX	Highly divergent homeobox	Unknown	None
APOOL	Apolipoprotein O like	Lipoprotein complexes circulation	None
SATL1	Spermidine/spermine N1-acetyl transferase-like 1	Unknown	None
ZNF711	Zinc finger protein 711	Similarity to protein that acts as a transcriptional activator	Mental retardation, X-linked type 97
POF1B	Premature ovarian failure, 1B	Germ cell division	Premature ovarian failure
CHM	Rab escort protein 1	Regulation of vesicular traffic	Choroideremia
DACH2	Dachshund family transcription factor 2	Regulation of organogenesis and myogenesis	None
KLHL4	Kelch like family member 4	Banding domain of actin	None
CPXCR1	CPX chromosome region, candidate 1	Contains a motif similar to motifs in zinc finger protein	None

## Discussion

Patients with choroideremia, deafness, and mental retardation were first described by Ayazi [1]. In a kindred (XL-45), two brothers and their maternal uncle exhibited this syndrome, while the females had only characteristic retinal changes. The second kindred (XL-62) was reported by Nussbaum [2], in which two matrilineal first cousins had choroideremia, mental retardation, deafness, and short stature. Nussbaum found that affected members from both kindreds, XL-45 and XL-62, carried similar size deletions in Xq21. Following fine mapping studies revealed that the deleted region contained putative loci for choroideremia, X-linked deafness-2 (DFNX2), and non-specific X-linked mental retardation. These loci are currently known as genes *CHM* and *POU3F4* [5]. Another case was reported by Song et al. [3], in which a 3-year-old boy showed deafness and mild mental retardation, while genetic analysis confirmed the presence of a 16 Mb Xq21 deletion. In this kindred (SV-08-20), the carrier mother had only mild high-tone hearing loss. To the best of our knowledge, so far, our patient is the seventh reported case with this syndrome. Although the genetic deletion and clinical phenotype are highly typical compared to the previous reports, there are still notable distinctions. For instance, in our case the patient’s mother exhibits no symptoms, which is consistent with most of the female carriers in XL-62 but not with those in XL-45. A detailed comparison of all the seven cases and female carriers is made in Table 2.

**Table 2:** Detailed comparison of the phenotype, the deleted region and the genes included in the probands and female carriers in the reported kindreds

Kindred	Phenotype of male patients	Phenotype of female carriers	Deleted region in Chr X	Refered OMIM genes	Reference
XL-45	Choroideremia, mental retardation, deafness	Retinal changes of choroiderenia	Between DXS232 and DXS95 (fine mapping) DXS232 and DXS95 DXS232 and DXS95	Not reported	Ayazi,1981 Nussbaum,1987
XL-62	Choroideremia, short stature, mental retardation, deafness	Mild high frequency sensorineural hearing loss (seen in only one out of four)	Between DXS72 and DXS214 (fine mapping) DXS232 and DXS95 DXS232 and DXS95	Not reported	Nussbaum, 1987
SV-08-20	Severe bilateral hearing loss, central hypotonia, developmental delay, mild mental retardation, vesicoureteral reflux	Mild high-tone hearing loss	16 Mb Xq21 (estimated by PCR)	*POU3F4, CHM*	Song, 2010
Present case	Choroideremia, mental retardation, hearing impairments, cochlea deformity, facial anomalies	Normal	8.05 Mb Xq21 Chr X: (80,817,978-88,868,979)	*CHM, POU3F4, ZNF711, POF1b*	-

As deafness and choroideremia are commonly observed in all the recorded cases, *POU3F4* and *CHM* gene deletions are two basic indexes for diagnosis. *POU3F4* gene encodes POU-III class factor 4, a transcription factor that mediates epigenetic signals, which induce striatal neuron-precursor differentiation. By generating non-functional products, mutations in this gene often lead to DFNX2, which is characterized by conductive and sensorineural progressive hearing loss and pathognomonic temporal bone deformity, including dilatation of the inner auditory canal and fistulous connection between the internal dilatation auditory canal and the cochlear basal turn [6]. Accordingly, our patient showed inner ear deformity with short base turn of the cochlea, unclear division within the cochlea and the absence of the modiolus, which is a typical consequence of *POU3F4* deletion.

*CHM* gene defect is a confirmed cause of choroideremia, an X-linked disease leading to progressive vision loss. *CHM* encodes REP1, a subunit of geranylgeranyl transferase that affects the regulation of vesicular traffic. Deletions and point mutations in *CHM* generate a truncated product and result in degeneration of the choriocapillaris, retinal pigment epithelium, and eye photoreceptors [7]. Despite the strong pathogenicity in male patients, mutations in *CHM* do not have the same effect on female carriers. This might be a consequence of the non-random inactivation of the X chromosome, which has been validated by Carrel and Willard in their studies on *CHM-*inactivation escaping pattern [8].

Notably in previous reports, all male probands exhibited prominent phenotypes, including both choroideremia and hearing impairment, while the female carriers of each kindred exhibited only one of the clinical characteristics and their symptoms were milder. In our case, the mother manifests no symptoms, similar to the female carriers in kindred XL-62. This diversity of female carrier’s phenotypes shows that although a regressive inheritance pattern is clear, the exact influence of gene loss towards female carriers is still unpredictable.

*ZNF711* is a member of the zinc-finger gene family. It has been proved that the ZNF711 protein interacts, as a cofactor, with demethylase PHF8 in binding to target genes, thus suggesting a role for *ZNF711* in transcriptional modulation [9]. Although limited in clinical case reports, *ZNF711* gene loss is believed to be causative to non-specific X-linked mental retardation(MRX97). By exon sequencing of the X chromosome in 208 families with X-linked mental retardation, Tarpey et al. 2009. identified two truncating mutations of *ZNF711* in two families with MRX97 [10]. Clinical reports of sporadic cases also support this pathogenetic connection [11]. Recently in 11 male patients from two new found MRX97 families, Van der Welf et al. [12] identified a frameshift deletion and a missence mutation in *ZNF711* which both predict the deleterious effect and cause the disease. By expression analysis of cell culture, they also showed that these mutations induced differential expression of genes known to be expressed in the brain compared to controls, which adds evidence to *ZNF711* as a transcription factor. In our case, our patient exhibits severe intellectual disabilities and facial dysmorphism, which are coincided with the phenotype of MRX97. Considering of other pathogenetic genes (*POU3F4* and *CHM*) have no independent effect to mental retardation, *ZNF711* loss may have contributed majorly to the MRX97 phenotype of our patient.

**Fig. 4:**
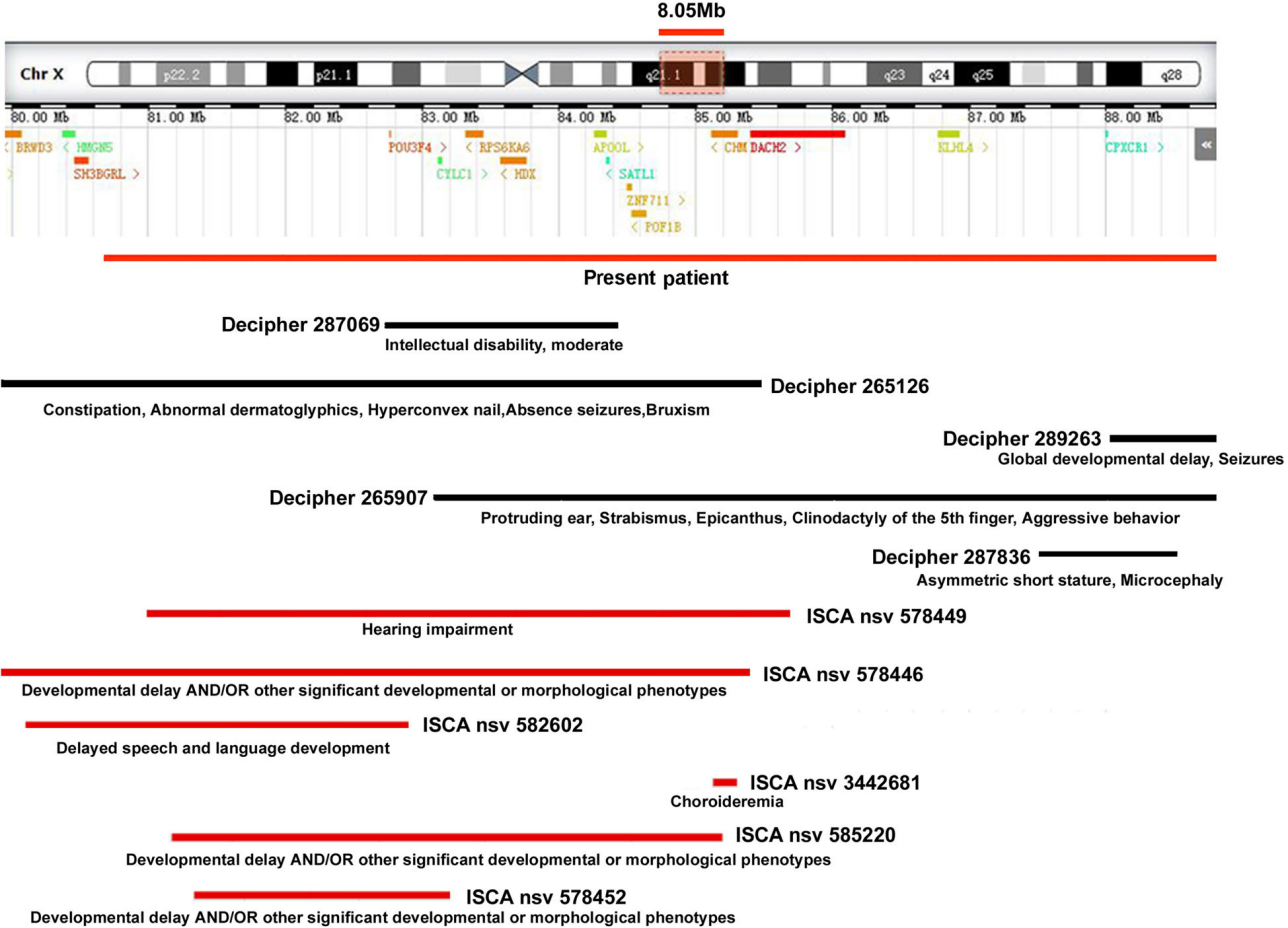
Xq21.1 deletion. The upper part of the figure illustrates the exact locations of the protein coding genes included in this region. The lower part of the figure schematically represents the Xq21.1 deleted region in our patient(in *red*) and other deletions reported in the Decipher(in *black*) and ISCA database(in *red*). The ID of each patient is reported. The clinical features of the patients are reported in parentheses below the corresponding deletion as described in Decipher (https://decipher.sanger.ac.uk/) and ISCA (https://www.clinicalgenome.org/)

We continued to search in the Decipher and ISCA databases for similar size deletions at Xq21.1 and identified 11 deletions with certain pathogenicity. Although most of them have concurrent phenotypes, none is exactly corresponds to the phenotypes of our patient. A notable fact is that deletions which does not contain *ZNF711* are often not associated with mental retardations (Decipher 289,263, 287,836, ISCA 582602, 3,442,681, and 578,452, Fig. 4) or only with moderate MD (Decipher 287,069, Fig. 4). This furtherly suggests that *ZNF711* gene loss might be an independent cause to mental retardation.

## Conclusion

In conclusion, our male patient carrying the 8.05 Mb Xq21 deletion exhibits a typical phenotype of choroideremia, deafness, and mental retardation syndrome, an association which has been demonstrated by previous cases. Meanwhile, as it is commonly observed in other female carriers, the mother of our patient, who carries the same deletion, is non-symptomatic. Additionally, comparing to previous reports our patient has a more compact deleted genome region which contains less OMIM genes. This may help to prune down the causative genes of disease phenotypes. Although our study confirms the causative effect of the Xq21 deletion in males with multiple congenital defects, more cases are needed to be studied in order to clarify the consequences of gene loss in this chromosomal region and the effects on the phenotype.

## Abbreviation

*APOOL*: Apolipoprotein O like
ChAS: Chromosome analysis suite (Affymetrix)
*CHM*: Rab escort protein 1
Chr: Chromosome
CPXCR1: CPX chromosome region, candidate 1
*CYLC1*: Cylicin, basic protein of sperm head cytoskeleton 1
*DACH2*: Dachshund family transcription factor 2
DFNX2: Deafness, X-linked 2
HDX: Highly divergent homeobox
HRCT: High-resolution computed tomography
IQ: Intelligence quotient
KLHL4: Kelch like family member 4
OMIM: Online Mendelian inheritance in man
PCR: Polymerase chain reaction
*POF1B*: Premature ovarian failure, 1B
*POU3F4*: POU class 3 homeobox 4
*RPS6KA6*: Ribosomal protein S6 kinase A6
*SATL1*: Spermidine/spermine N1-acetyl transferase-like 1
SNP: Single-nucleotide polymorphism
Xq21: X chromosome long arm q band 21
*ZNF711*: Zinc finger protein 711
